# Vitamin, antioxidant and micronutrient supplementation and the risk of developing incident autoimmune diseases: a systematic review and meta-analysis

**DOI:** 10.3389/fimmu.2024.1453703

**Published:** 2024-12-09

**Authors:** Chen Ee Low, Sean Loke, Nicole Shi Min Chew, Ainsley Ryan Yan Bin Lee, Sen Hee Tay

**Affiliations:** ^1^ Yong Loo Lin School of Medicine, National University of Singapore, Singapore, Singapore; ^2^ Division of Rheumatology and Allergy, Department of Medicine, National University Hospital, Singapore, Singapore

**Keywords:** autoimmune diseases, immunology, multiple sclerosis, nutrition, rheumatoid arthritis, systemic lupus erythematosus

## Abstract

**Background:**

Autoimmune diseases pose significant health challenges worldwide and affect millions. In recent years, there has been growing interest in exploring preventive strategies through nutritional interventions using vitamins, antioxidants, and micronutrients to reduce the risk of developing autoimmune diseases. However, excessive supplementation has also been associated with toxicity.

**Objective:**

We aim to assess how the intake of vitamins, antioxidants and micronutrients affect the risk of developing autoimmune diseases.

**Methods:**

This PRISMA-adherent systematic review involved a systematic search of PubMed, Embase and Cochrane for controlled studies that evaluated the risk of incident autoimmune diseases after supplementation. Random effects meta-analyses were used for primary analysis.

**Results:**

18 studies were included. Overall meta-analyses observed that vitamin D did not influence the risk of autoimmune diseases (RR=0.99, 95%CI: 0.81-1.20). However, among the different vitamin D dosages, subgroup analysis demonstrated that those who were supplemented with 600-800IU/day may have a statistically significant reduction in risk (RR=0.55, 95%CI: 0.38; 0.82). Systematic review suggested that consumption of most vitamins, micronutrients and antioxidants may not have any effect on the risk of autoimmune diseases. Smoking, age, physical or outdoor activity and diet were significant confounding factors that affected the efficacy of such interventions.

**Conclusion:**

We studied the effect of various vitamins, micronutrients and antioxidants on the risk of developing autoimmune diseases. Our study contributes to the evolving landscape of nutritional immunology, providing a foundation for future research to unravel more definite relationships with supplementation and the development of incident autoimmune diseases.

**Systematic review registration:**

https://www.crd.york.ac.uk/prospero/, identifier CRD42024504796.

## 1 Introduction

Autoimmune diseases represent a diverse group of conditions characterised by the immune system’s aberrant response against the body’s own antigens (1) due to loss of immune tolerance (2). These diseases pose significant health challenges worldwide, impacting millions (2, 3). In 2019, the Global Burden of Diseases, Injuries, and Risk Factors Study (GBD) estimated the global incidence of autoimmune diseases to be 67 million (4). Women are especially susceptible to autoimmune diseases, making up approximately 80% of the disease population (5, 6).

Traditional methods used to treat autoimmune diseases mainly consist of immunosuppressive medications that dampen the body’s immune responses against self (7). Despite these treatments, the prevalence of autoimmune and inflammatory diseases continues to rise. Studies estimate that the annual increases in the overall global incidence and prevalence of autoimmune diseases stand at 19.1% and 12.5%, respectively (8). Currently, no therapy can modulate or reprogram the development of any autoimmune diseases. It can be assumed that the current state of therapies is symptomatic, blocking inflammatory cytokines but leaving the prevention of incident autoimmune diseases largely untouched. Nonetheless, risk factors for autoimmune diseases have further been identified, including genetic factors (9) and lifestyle choices like smoking, stress (10) and physical activity (11).

This rise in incidence and prevalence of autoimmunity necessitates a deeper understanding of modifiable factors that may influence autoimmune disease risk. In recent years, there has been a growing interest in exploring preventive strategies beyond conventional treatments, through improved understanding of the immunological underpinnings of autoimmunity (12, 13). Emerging research suggests that nutritional interventions, specifically through supplementation, could have a pivotal role in altering the risk of developing autoimmune conditions (14, 15). Vitamins, in particular vitamin D (16), and antioxidants such as omega-3 fatty acids, have garnered attention for their potential anti-inflammatory effects (17). They are also postulated to have modulatory effects on the immune system (18). Research has shown that the vitamin D receptor is expressed on multiple cells in the immune system, such as monocytes, dendritic cells and activated T-cells (19). Studies have shown that this binding of vitamin D to its receptor on immune cells can inhibit pro-inflammatory activity by inhibiting T-lymphocyte proliferation and is hence associated with a decrease in pro-inflammatory cytokines interleukin 2 (IL-2) (20) and interferon gamma (IFN-gamma) (21). Vitamin D deficiency has been observed in patients with autoimmune diseases such as rheumatoid arthritis, Sjogren’s syndrome, systemic lupus erythematous and systemic sclerosis (22). Furthermore, low levels of vitamin D in these patients have been associated with poorer disease outcome and course (23, 24). On the other hand, excessive intake of vitamin supplements has also been associated with potential toxicity (25).

Dietary patterns rich in antioxidants such as vitamins are also believed to improve overall health and decrease oxidative stress, preventing disease (26). The Mediterranean Diet is one example, focusing on a healthy diet consisting of whole grains, fruits, vegetables, seafood and nuts. This diet has been postulated to have anti-inflammatory effects on the human body, potentially reducing the risk of autoimmune diseases (27). Significantly, a study conducted by Skoldstam et al. found that in a population of patients diagnosed with rheumatoid arthritis, intervention with the Mediterranean Diet obtained a substantive reduction in inflammatory activity, resulting in increased mobility and hence better quality-of-life overall (28). Obtaining a more comprehensive understanding of the components in such diets that can alter immune system activity will be crucial to our knowledge of the dietary factors that can modify the risk of developing autoimmune diseases.

As individuals increasingly turn to complementary approaches for health maintenance (29), using vitamins, antioxidants or micronutrients supplementation, it becomes imperative to rigorously assess the collective evidence supporting the potential risk of certain dietary supplements on the development of incident autoimmune diseases. We aim to consolidate existing literature to assess how the intake of vitamins, antioxidants and micronutrients affect the risk of developing autoimmune diseases.

## 2 Methods

We report our systematic review according to the Preferred Reporting Items for Systematic Reviews and Meta-Analyses (PRISMA) guidelines. Our protocol was registed on PROSPERO (CRD42024504796).

### 2.1 Search strategy

Literature search was performed in PubMed, Embase, and Cochrane. Our search strategy combined terms for vitamins, antioxidants, micronutrients, and autoimmune diseases. The database-controlled vocabulary was used to search subject headings. A spectrum of synonyms with appropriate truncations was used to search title, abstract, and author keywords. The search strategy was translated between the databases. Examples of the search strategies for PubMed and EMBASE are available in **Supplementary Table S1**.

### 2.2 Inclusion and exclusion criteria

**Figure 1:**
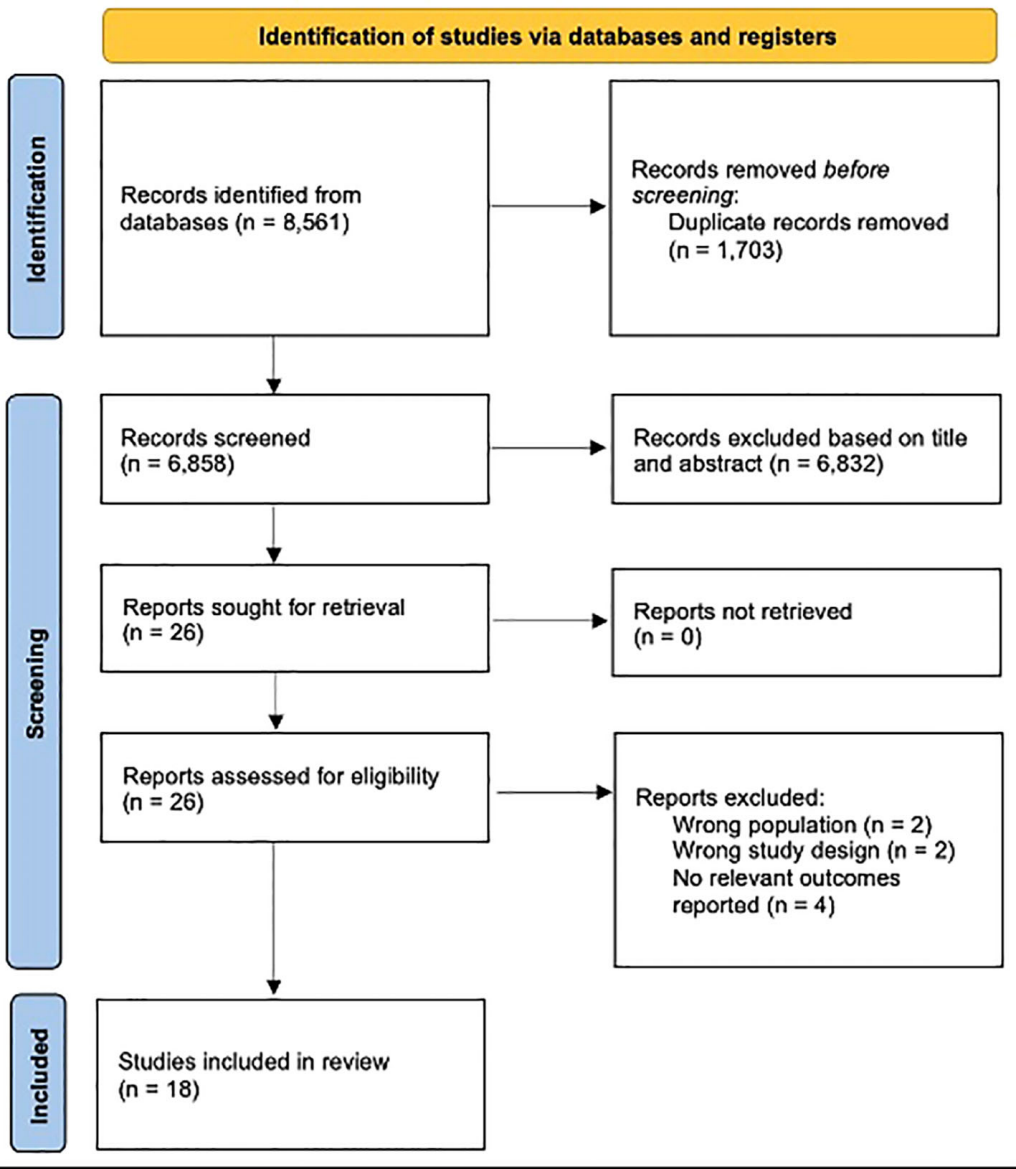
PRISMA flowchart.

Two reviewers independently screened titles and abstracts of all studies for eligibility. The full text of studies assessed as ‘relevant’ or ‘unclear’ was then independently assessed by a third reviewer. All peer-reviewed English-language studies published since 2000 that evaluated the risk of autoimmune diseases following the supplementation of vitamins, antioxidants or micronutrients were included. Non-empirical studies, grey literature, studies without a control-arm and abstracts were excluded. The selection process is shown in **Figure 1**.

### 2.3 Data extraction

Two reviewers independently performed the extraction with quality checking performed at the end. Subject matter information included the aim of the study, demographics, and characteristics of control group, and main findings of the study.

### 2.4 Statistical analysis

We conducted all analyses on R (version 4.1.0) using the *meta* and *metafor* packages. A two-sided P value of <0.05 was considered as statistically significant unless specified. Studies were pooled for meta-analyses using the relative risk of the autoimmune disease [measured using risk ratios (RR) compared to controls]. Sensitivity analysis was conducted using identification and exclusion of potential outliers and the leave-one-out analysis. Between-study heterogeneity was represented using I2 and τ2 statistics. I2 of <30% demonstrated low heterogeneity between studies, 30% to 60% revealed moderate heterogeneity, and >60% showed substantial heterogeneity (30). We performed subgroup analyses and meta-regression to determine if any key categorical and hierarchical variables influenced the results. We assessed for publication bias quantitatively using Egger’s test. Visual inspection for funnel plot asymmetry was used for qualitative publication bias. If we suspected publication bias, sensitivity analysis was conducted using the trim-and-fill method (R0 estimator, fixed-random effects models) to estimate the pooled effect size after imputing potential studies (31). If publication bias was absent, this assumes a normal distribution of effect sizes around the center of the funnel plot (32).

### 2.5 Risk of bias

Two independent reviewers assessed for the methodological quality and risk of bias of the included studies using the Joanna Brigg’s Institute (JBI) Critical Appraisal tool (33). Any discrepancies were resolved by a third reviewer.

## 3 Results

### 3.1 Characteristics of included studies

A total of 18 studies (17, 34–50) were included from 8,561 records (**Figure 1**). The remaining 8,543 studies were excluded after removing irrelevant studies with the wrong study design, population, outcomes, and the duplicates. Eight studies reported on multiple sclerosis (34–36, 38, 40, 43, 46, 47), eight studies reported on rheumatic arthritis (39, 41, 42, 44, 45, 48–50), two studies reported on systemic lupus erythematosus (48, 49) and two studies did not report the specific autoimmune disease (17, 37). The exposures included vitamin A (38, 41, 48), B (38, 41, 44, 46), C (38, 39, 41, 46, 48), D (17, 34–36, 38, 40–42, 44, 45, 49), E (37, 39, 41, 44, 48), multivitamins (41, 46), iron (38, 40, 41, 44, 46), calcium (38, 41, 45, 47), omega-3 (17, 41, 46), zinc (38, 39, 41, 44, 47), dietary antioxidants (38, 50). Among 945,471 participants, 4,591 patients developed autoimmune diseases. Main characteristics of the included studies can be found in **Table 1**.

**Table 1:** Overall characteristics.

Author	Publication year	Region of study	Gender male (%)	Mean age (SD)	Characteristics of the controls	Proportion of smoking (%)	Autoimmune disease	Exposures	Number at risk (exposed)	Number of events (exposed)	Number at risk (control)	Number of events (control)
Dehghan (34)	2018	Iran	23.5	30.9 (3.51)	Matched healthy	14.2	Multiple Sclerosis	Vit D	120	46	360	98
Butzkueven (35)	2023	Australia	28.6	37.0 (10.3)	Placebo	14.6	Multiple Sclerosis	Vit D	49	25	50	27
Cavalla (36)	2022	Italy	29.0	35.4 (9.36)	Matched healthy	NR	Multiple Sclerosis	Vit D	83	NA	83	NA
Hahn (17)	2021	USA	49.4	67.1 (7.1)	Placebo	7.2	All	Vit D, Omega-3	12927	123	12944	155
Karlson (37)	2008	USA	0.0	54.6 (7.0)	Placebo	49.0	All	Vit E	19576	50	19568	56
Abdollahpour (38)	2022	Iran	41.1	30.8 (8.2)	Matched healthy	NR	Multiple Sclerosis	Vit D, Vit A, Vit B6, Vit C, Calcium, Iron, Zinc, Dietary Antioxidant Index	547	NA	1057	NA
Cerhan (39)	2003	USA	0.0	61.4 (NR)	Nil	33.0	Rheumatoid Arthritis	Vit C, Vit E, Zinc	29368	152	NA	NA
Cortese (40)	2015	Norway	27.9	45.6 (10.7	Matched healthy	52.0	Multiple Sclerosis	Vit D, Iron	953	79	1717	160
Kronzer (41)	2022	USA	30.0	64.0 (NR)	Matched healthy	44.0	Rheumatoid Arthritis	Vit D, Vit A, Vit B, Vit C, Vit E, multivitamin, Calcium, Omega-3, Iron, Zinc	212	159	636	482
Merlino (42)	2004	USA	0.0	61.5 (4.2)	Nil	33.0	Rheumatoid Arthritis	Vit D	29368	152	NA	NA
Munger (43)	2011	USA	0.0	39.9 (NR)	Matched healthy	46.0	Multiple Sclerosis	Vit D	121024	33	121024	318
Pedersen (44)	2005	Denmark	48.0	57.0 (7.0)	Nil	NR	Rheumatoid Arthritis	Vit D, Vit E, Vit B, Iron, Zinc	56691	69	NA	NA
Racovan (45)	2011	USA	0.0	62.3 (6.92)	Placebo	7.5	Rheumatoid Arthritis	Vit D, Calcium	16238	83	16197	80
Rezaeimanesh (46)	2021	Iran	22.0	37.2 (10.5)	Matched healthy	NR	Multiple Sclerosis	Vit B12, Vit C, Omega-3, Multivitamin, Iron	143	2	400	51
Cortese (47)	2019	Iran	0.0	NR	Nil	NR	Multiple Sclerosis	Zinc, Calcium	175431	479	NA	NA
Costenbader (48)	2010	USA	0.0	NR	Nil	NR	Rheumatoid Arthritis and Systemic Lupus Erythematosus	Vit A, Vit C, Vit E	184643	737	NA	NA
Hiraki (49)	2010	USA	0.0	NR	Nil	NR	Rheumatoid Arthritis and Systemic Lupus Erythematosus	Vit D	119173	976	NA	NA
Moradi (50)	2022	Iran	21.9	43.7 (11.46)	Matched Healthy	7.1	Rheumatoid Arthritis	Dietary Antioxidant index	100	NA	197	NA

NR, Not reported; NA, Not available.

**Figure 2:**
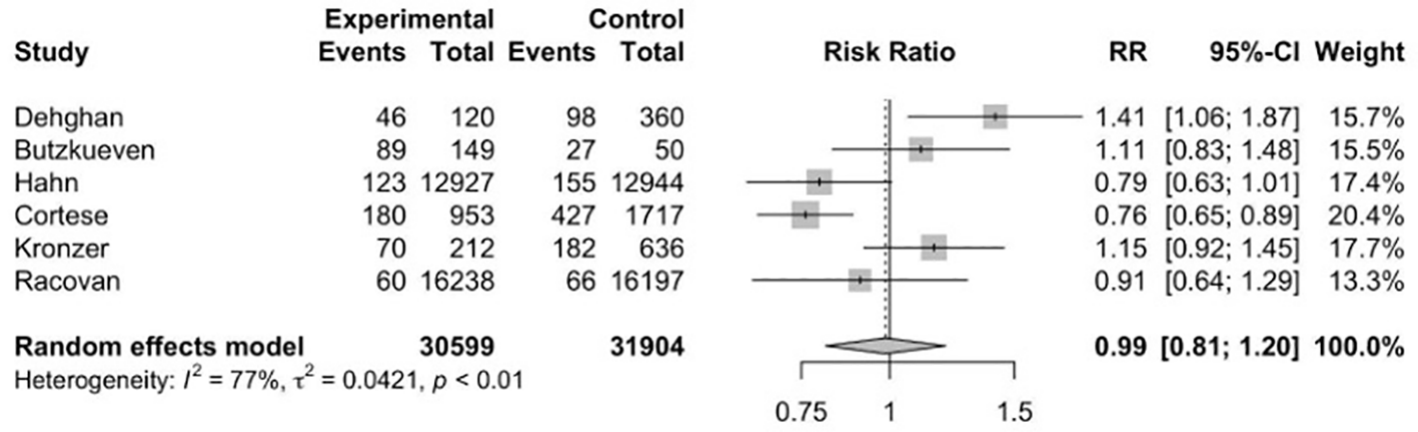
Relative risk ratio of Vitamin D supplementation and autoimmune diseases. RR, risk ratio; CI, confidence interval.

**Figure 3:**
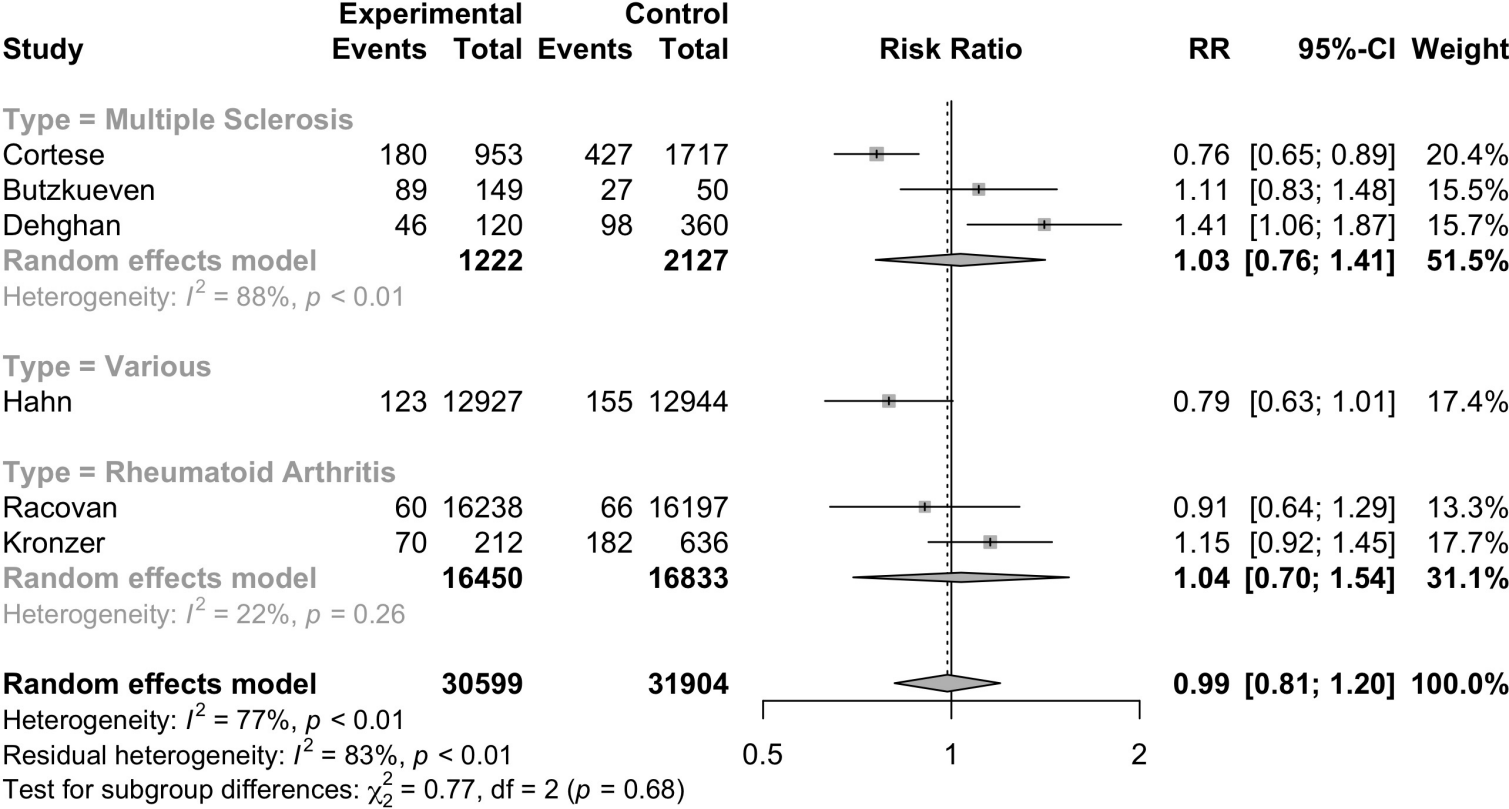
Relative risk ratio of Vitamin D supplementation and autoimmune diseases stratified based on disease type. RR, risk ratio; CI, confidence interval.

Six studies (17, 34, 35, 40, 41, 45) were included to evaluate the RR of autoimmune diseases amongst those with vitamin D supplementation. Meta-analysis of the six studies (**Figure 2**) indicated that vitamin D supplementation does not seem to affect the risk of autoimmune diseases (RR=0.99, 95%CI: 0.81-1.20). Subgroup analyses showed that those who consumed both dietary sources and supplementary vitamin D may have significant risks of developing autoimmune diseases (RR=1.26, 95%CI: 1.00-1.58) as compared to those who consumed supplementary vitamin D alone (RR=0.85, 95%CI: 0.73-0.99) (p<0.01) (**Table 2**). Subgroup analyses showed that the type of autoimmune disease did not significantly affect the risk of developing autoimmune diseases after consumption of supplementary vitamin D (RR=0.99, 95%CI: 0.81-1.20) (**Figure 3**). Other categorical variables such as country of study, control type, gender, age, study design, and smoking were also not found to significantly reduce the risk of autoimmune diseases (**Table 2**). Meta-regression was also insignificant (**Supplementary Table S2**).

**Table 2:** Subgroup meta-analyses of vitamin D supplementation and relative risk ratio of autoimmune disorders using the random effect model.

Variable	Cohorts	Number at risk (Exposed)	Number at risk (Controls)	Risk Ratio	95% CI	I2	Test of interaction (p-value)
Overall	6	30599	31904	0.99	0.81; 1.20	77%	NA
Control = Matched	3	1285	2713	1.05	0.79; 1.39	89%	0.55
Control = Placebo	3	29314	29191	0.92	0.68; 1.25	34%	
Country = Iran	1	120	360	1.41	1.06;1.87	NA	0.12
Country = Australia	1	149	50	1.11	0.83;1.48	NA	
Country = USA	3	29377	29777	0.95	0.75;1.19	61.0%	
Country = Norway	1	953	1717	0.76	0.65;0.89	NA	
Gender = <25%	2	16358	16557	1.15	0.80;1.65	73.0%	0.32
Gender = ≥25%	4	14241	15347	0.93	0.73;1.17	75.0%	
Design = Case control	3	1285	2713	1.05	0.79;1.39	89.0%	0.55
Design = RCT	3	29314	29191	0.92	0.68;1.25	34.0%	
Age = <50	3	1222	2127	1.03	0.77;1.39	88.0%	0.68
Age = >50	3	29377	29777	0.94	0.70;1.28	61.0%	
Intake = Both	2	332	996	1.26	1.00;1.58	13.0%	**<0.01**
Intake = Supplement	4	30267	30908	0.85	0.73;0.99	45.0%	
Type = MS	3	1222	2127	1.03	0.76;1.41	88.0%	0.68
Type = RA	2	16450	16833	1.04	0.70;1.54	22.0%	
Type = Various	1	12927	12944	0.79	0.63;1.01	NA	
Smoking = <30%	4	29434	29551	1.03	0.79;1.34	70.0%	0.64
Smoking = >30%	2	1165	2353	0.92	0.65;1.31	89.0%	

NA, Not Available; NR, Not Reported; MS, Multiple Sclerosis; RA, Rheumatoid Arthritis.

**Figure 4:**
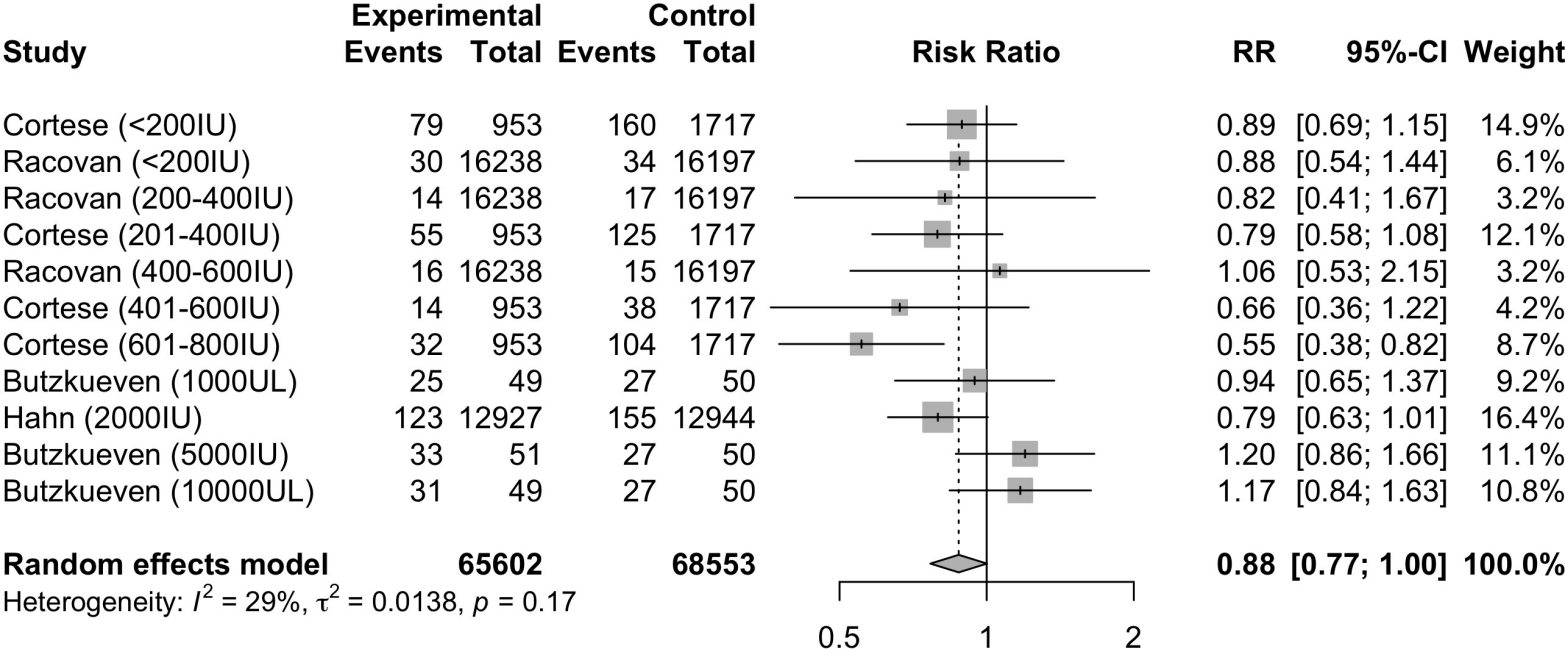
Relative risk ratio of Vitamin D supplementation and autoimmune diseases stratified based on studies with reported dosage. RR, risk ratio; CI, confidence interval.

Four studies (17, 35, 40, 45) evaluated the RR of autoimmune diseases based on the dosage of vitamin D supplementation. After stratification by dosage, the individual cohorts were reported separately by the studies. Meta-analysis of the 11 cohorts (**Figure 4**) indicated that the dosage of vitamin D supplementation may reduce the risk of autoimmune diseases, although borderline statistically insignificant (RR=0.88, 95%CI: 0.77-1.00). Subgroup analysis (**Supplementary Table S3**) of the dosage of vitamin D supplementation suggested a statistically significant reduction in risk of autoimmune diseases among those who were supplemented with vitamin D dosages of 600-800IU/day (RR=0.55, 95%CI: 0.38; 0.82). Subgroup analyses of other categorical variables were not performed due to the possibility of type 1 error.

### 3.2 Other vitamins, antioxidants or micronutrients

Meta-analyses of supplementation with other vitamins, antioxidants or micronutrients can be found in **Supplementary Table S4** and revealed that supplementation with vitamin C, B, multivitamin, iron, omega-3 demonstrated a statistically insignificant decrease in risk of autoimmune diseases. Only supplementation with vitamin E demonstrated a statistically insignificant increase in risk of autoimmune disease (RR=1.17, 95%CI: 0.65; 2.10).

Due to the limited number of studies of each vitamin, antioxidant and micronutrient, systematic review was additionally performed and reported (**Supplementary Tables S5, S6**).

### 3.3 Multivitamins and vitamin A, B, C, E

Three studies reported that vitamin A had none (41, 48) to significant reduction (38) in autoimmune diseases. Vitamin A supplementation was shown to reduce risk of multiple sclerosis (38) but not in rheumatoid arthritis (41, 48).

Four studies reported that vitamin B had none (41, 44) to significant reduction (38, 46) in autoimmune diseases. Both Rezaeimanesh et al. (46) and Abdollahpour et al. (38) demonstrated reduction in risk of developing multiple sclerosis as an adult after consuming vitamin B during adolescence while the other two studies investigated the incidence in older adults.

Five studies reported that vitamin C had none (41, 48) to significant reduction (38, 39, 46). Vitamin C supplementation was shown to reduce risk of multiple sclerosis (38, 46) but not in rheumatoid arthritis and systemic lupus erythematosus (41, 48).

All five studies on vitamin E (37, 39, 41, 44, 48) and two studies on multivitamins (41, 46) did not find any significant effect on the risk of autoimmune diseases.

### 3.4 Micronutrients and antioxidants

Three studies reported that omega-3 had none (41) to significant reduction (36, 40). Cortese et al. (40) showed reduction in risk of developing multiple sclerosis in adulthood with intake of cod liver oil containing omega-3 during adolescence. Cavalla et al. (36) found that the dietary omega 6/omega 3 polyunsaturated fatty acids ratio was higher in patients than in controls, causing reduction in risk.

Four studies on calcium reported none (41, 45, 47) to significant reduction (38). This is possibly related to age, with the younger population having reduced risk (38) while this correlation was not observed in the older populations (41, 45, 47). Interestingly, Kronzer et al. (41) reported that while there was no correlation between intake of supplemental calcium and the risk of rheumatoid arthritis, dietary calcium intake of two to three times a day was associated with a reduced risk of rheumatoid arthritis.

Five studies on zinc reported none (41, 44, 47) to significant reduction (38, 39). Cortese et al. (47) reported that women with the highest total zinc intake and highest intake of supplemental zinc-including multivitamins showed a reduced risk of multiple sclerosis, however no correlation was found between intake of zinc-only supplements. Cerhan et al. (39) reported that intake of supplemental zinc was associated with a reduction in risk of developing rheumatoid arthritis, while intake of dietary zinc was associated with an increased risk of developing rheumatoid arthritis.

Five studies on iron (38, 40, 41, 44, 46) and two on dietary antioxidants index (38, 50) did not observe any significant effect in risk of developing autoimmune diseases.

### 3.5 Diet

Six (34, 36, 38, 42, 44, 46) out of seven studies (34, 36, 38, 41, 42, 44, 46) reported a significant association between diet and risk of autoimmune diseases (**Supplementary Table S7**). Three (38, 42, 46) out of the six studies saw a significant reduction in risk of autoimmune diseases while the other three (34, 36, 44) saw a significant increase in risk. Abdollahpour et al. (38) showed a reduced risk of multiple sclerosis with egg, red meat, poultry and dietary supplement intake. Rezaeimanesh et al. (46) demonstrated that more intake of dairy, seafood, red meat, vegetable, fruit and nut during adolescence resulted in reduction in risk of multiple sclerosis in adulthood. Merlino et al. (42) revealed that an intake of more than 68 servings of milk products in a month reduced risk of developing rheumatoid arthritis. Deghan et al. (34) suggested that a carnivorous diet increased risk of multiple sclerosis when compared to a vegetarian diet. Pedersen et al. (44) found that consuming medium fat fish containing 3-7grams of fat per 100 grams was associated with increased risk of rheumatoid arthritis. Cavalla et al. (36) saw that more intake of rapid absorption carbohydrates, lesser vegetal proteins, and more animal proteins were observed in patients with a first demyelinating event.

### 3.6 Demographical characteristics

Four studies (17, 34, 35, 45) reported a significant association between personal characteristics variables and risk of autoimmune diseases (**Supplementary Table S8**). Two studies (35, 45) reported that the risk significantly increases with age. One study (34) evaluated that those with lower income had a higher risk. Hahn et al. (17)showed a significantly increased efficacy of omega-3 fatty acids in reducing risks among those with a family history of autoimmune diseases.

### 3.7 Smoking status

Three studies (36, 38, 44) investigated the association between smoking and risk of autoimmune diseases (**Supplementary Table S9**). Abdollahpour et al. (38) found a significantly increased risk of developing multiple sclerosis with lifetime second-hand smoking. Pedersen et al. (44) reported a significantly increased risk of developing rheumatoid arthritis for current smokers as compared to former smokers. Cavalla et al. (36) showed an increased risk of developing a first myelinating event in someone who had ever smoked.

### 3.8 Physical or outdoor activity

Three studies (34, 38, 41) revealed a significant association between physical or outdoor activity and reduced risk of autoimmune diseases (**Supplementary Table S10**). Interestingly, Abdollahpour et al. (38) demonstrated that the risks were reduced the most in those with more than five hours of sunlight exposure daily.

### 3.9 Risk of bias and publication bias

We scored for the quality of the methodology of the 18 studies using the JBI checklist and presented the results in **Supplementary Table S11**. Overall, there was no significant risk of bias identified. Sensitivity analyses using funnel plots, trim-and-fill, Egger’s test showed some publication bias (**Supplementary Figures S1–S3, S5**). However, leave-one-out analyses showed no singular studies that would affect the overall results (**Supplementary Figure S4**).

## 4 Discussion

To the best of the authors’ knowledge, this is the first systematic review and meta-analyses to examine the relationship between supplemental vitamins, micronutrients, and antioxidants on the risk of autoimmune diseases. This systematic review and meta-analysis sought to investigate any modifiable risk of developing autoimmune diseases with dietary consumption of various vitamins, micronutrients, and antioxidants. Our results demonstrated that vitamin D supplementation may not have any effect on modifying the risk of developing autoimmune disease. However, subgroup analysis by dosage of vitamin D showed that the dosage consumed at 600-800IU may reduce the risk of autoimmune diseases. Those who consumed both dietary and supplementary vitamin D had a higher risk of autoimmune disease as compared to those on supplementation alone. Systematic review suggests that consumption of most vitamins, micronutrients and antioxidants may not have any effect on the risk of autoimmune diseases. Smoking, age, physical or outdoor activity, and diet were significant factors that affected the efficacy of such interventions.

Current literature suggests that vitamin D levels had been most closely associated with modifying the risk of developing multiple sclerosis compared to other autoimmune diseases (51). A landmark investigation by Munger et al. (52) determined that higher concentrations of serum 25-hydroxyvitamin D (25(OH)D) levels lead to a decreased risk of developing multiple sclerosis. The VITAL randomised controlled trial (17) further validated the potential role of vitamin D supplementation in significantly reducing the risk of autoimmune diseases. Despite this, it is pertinent to recognise that many lifestyle and physiological factors are involved in altering normal serum 25(OH)D levels (53), and supplementation with oral cholecalciferol does not linearly increase serum 25(OH)D levels (53). Hence, a fixed dose of vitamin D supplementation may not apply the same risk reduction in a cohort of subjects with varying characteristics. Furthermore, our study investigated different autoimmune diseases which exert vastly different systemic physiological changes (54) and possibly altered individuals’ response to vitamin D supplementation across different cohorts (55).

Vitamin D plays a key role as a hormone in regulating the immune system and influencing immune responses by functioning as a pro-survival molecule, protecting cells from harmful signals by suppressing inflammatory reactions (56). It does so by modulating pathways that affect the differentiation of T-helper-2 cells, M2 macrophages, and regulatory T cells, thereby helping to maintain immune homeostasis through the promotion of a tolerogenic state (56). In our review, most of the autoimmune diseases reported by the different studies were on multiple sclerosis and rheumatoid arthritis. Multiple sclerosis is characterised by an autoimmune response that targets the myelin sheath that insulates nerve fibres, leading to demyelination and impairing the ability of neurons to transmit electrical signals efficiently (57). Over time this repeated demyelination causes the scar tissue formation, leading to axonal damage further impairing neurological function (57). Although the exact pathophysiology of how vitamin D supplementation reduces risk of developing multiple sclerosis has yet to be elucidated, certain mechanisms have been proposed (58). For example, proteomic analysis revealed that after high dose vitamin D supplementation, 125 proteins were differentially regulated in the brains of 1,25(OH)2D-treated mice during remyelination, compared to placebo (59). Those upregulated proteins were primarily associated with calcium binding and mitochondrial function (59). Rheumatoid arthritis is a systemic inflammatory autoimmune disorder in which both T and B lymphocytes play a key role in pathogenesis (60). Vitamin D supplementation can influence both T and B lymphocyte populations, helping to regulate the immune response needed to prevent or manage the disease (61, 62).

While there is evidence that vitamin D supplementation can reduce the risk of autoimmune diseases, the high degree of variability between participants’ response to supplementation may explain the insignificant results. Genetic variants have been linked to the high variability in the efficacy of vitamin D supplementation (63). For instance, Ammar et al. reported a 18.8% in response variability among three single nucleotide polymorphisms post supplementation (63). Higher body mass index has also been shown to reduce supplementation efficacy in a subset of 16515 patients in the VITAL trial (64). Considering these variations in response can help optimise vitamin D supplementation to achieve a better response. Another explanation could be the studies having a lack of proper control group for vitamin D interventional studies as it is not ethically possible to keep individuals on long-term vitamin D deficiency. However, subgroup analysis suggested that vitamin D dosages of 600-800 IU/day significantly reduced the risk of autoimmune diseases. Cortese et al. described a strong protective effective of vitamin D 600-800 IU/day using cod liver during winter but not in other seasons for Norwegian adolescents developing incident multiple sclerosis during adulthood (40). This finding potentially underscores the importance of considering not just dosage variability but also seasonality when assessing the impact of vitamin D supplementation on autoimmune disease risk. We also noted a bimodal pattern of vitamin D supplementation when <200 IU/day or ≥5000 IU/day (**Supplementary Table S** **3**), with extremes of doses leading to paradoxically higher incident autoimmune diseases. This observation mirrors the findings of Lim et al., where a bimodal influence of vitamin D inducing inflammatory responses and fungal burden was observed in a mouse model of candidiasis (65). Lately, Carlberg et al. (66) has put forth this concept of a vitamin D response index that clusters the population to high, mid and low responders based on their transcriptome changes after supplementation. Further research should explore optimal dosages and interventional trials (e.g., N = 1 approach) tailored to specific populations, seasons and disease profiles, considering the multifaceted nature of both vitamin D metabolism and autoimmune pathophysiology (66).

Diet has been well alluded to as a possible modifiable risk factor of autoimmune diseases (67). Specifically, diets characterised by an abundance of fat and sodium, typified by the Western dietary pattern, have been observed to amplify systemic inflammation and exert negative effects on immune responses (68). Conversely, the Mediterranean diet has exhibited its potential in mitigating chronic inflammation (69). This dietary approach has also demonstrated its potential protective role against multiple sclerosis (67). Interestingly, Barrea et al. (70) demonstrated that the Mediterranean diet has the potential to significantly increase serum 25(OH)D levels in a cohort of 617 Caucasians. Vitamin D intake can be derived from specific dietary sources, notably oily fishes, and certain nuts (71), which are often found within a Mediterranean diet (72). While these factors suggest a potential scenario wherein dietary vitamin D may mitigate the risk of autoimmune diseases, our subgroup analyses unveiled a contrasting observation; individuals concurrently utilising both supplemental and dietary sources of vitamin D may have a higher risk of developing autoimmune diseases. Notably, one study included in our analysis (41) reported a prevalent consumption of fatty foods, poultry, and dairy among participants, while another study (34) reported a varied dietary pattern among its participants. We hypothesise that this observation could be attributed to the broader impact of an individual’s overall dietary patterns leading to high doses of vitamin D from supplementation and food intake, which may exert a more substantial influence than singular vitamin D supplementation in shaping the risk of autoimmune disease development. This is also in keeping with the bimodal response of vitamin D in preventing incident autoimmunity described above (**Supplementary Table S3**). More thorough interventional studies should be conducted to investigate the comparative effects of specific dietary patterns and micronutrients on the development of autoimmune disease.

While vitamin D stands as the most extensively investigated vitamin in relation to its role in modulating autoimmunity and the development of autoimmune diseases, our findings and systematic review also highlight the potential contributions of other vitamins, micronutrients, and antioxidants in reducing the risk of autoimmune diseases. Vitamin C has been well established as an effective antioxidant with immunoregulatory effects (73). Similarly, vitamin B, particularly vitamin B6, has established anti-inflammatory roles (74). Beyond vitamins, supplements like omega-3 are accruing evidence for their capacity to decrease morbidity and mortality associated with various autoimmune diseases (75), owing to pro-anti-inflammatory and immunoregulatory effects (76). The VITAL trial, while not reaching statistical significance, reported a 15% reduction in the risk of autoimmune diseases with omega-3 supplementation (17). Intriguingly, the direct impact of serum iron or ferritin supplementation on immune regulation or protection against immune diseases was not well studied. However, existing literature suggests that disturbances in physiological iron metabolism may potentially influence individual immunity (77). Like other vitamins, vitamin E has been demonstrated to exhibit immunoregulatory effects, especially for infectious disease (78). However, our findings did not demonstrate any significant reduction in risk of developing autoimmune diseases with vitamin E supplementation. While our meta-analysis primarily focused on the correlation between vitamin D supplementation and autoimmune disease risk, the inclusion of other vitamins, micronutrients and antioxidants adds complexity to our findings. Limited data for these factors underscores the need for caution in drawing definitive conclusions. Our study opens avenues for future research to build upon by expanding sample sizes and employing longitudinal designs to better understand temporal relationships. Stratified analyses and exploration of interactions among micronutrients may reveal nuances not captured in our study. Rigorous randomised controlled trials with defined dosages and consideration of confounding factors would further enhance causal inference.

This study also revealed a noteworthy correlation between specific demographic factors and modifying the risk of developing autoimmune diseases. Older age, smoking status and lack of physical or outdoor activity were found to be significant risk factors for autoimmune diseases. While most of the autoimmune diseases we investigated have a peak age of onset of less than 60 years old (79), older age consistently remains a significant risk factor for developing autoimmune diseases (80) and other pathologies (81–83). Similarly, smoking has been a well-established risk factor for autoimmune diseases (84). On the contrary, we noted a significantly reduced risk of developing autoimmune disease with increased physical outdoor activity. On top of the direct benefits of physical activity on immunoregulation (85) and reducing systemic inflammation (86), outdoor activities in areas with sufficient sunlight exposure has been shown to be a protective factor against multiple sclerosis, vis-à-vis via endogenous vitamin D production (87).

Our study should be interpreted in due consideration of the limitations. Firstly, we anticipated high heterogeneity in the reporting of outcomes. Data on the development of autoimmune diseases with vitamin D supplementation was only available for six studies. We were also unable to account for the different subgroups of vitamin D responders. The type of autoimmune disease was also different for each study, further contributing to this heterogeneity. However, we were able to perform subgroup analyses based on the type of autoimmune diseases. Thirdly, not all studies had consistent doses of the supplementation within the intervention group, hence we are unable to quantify the true dose-dependent extent of each supplementation’s role in risk modification of developing autoimmune disease. However, we were still able to analyse specific dose ranges in our subgroup and mixed-effect meta regression analysis.

## 5 Conclusion

In conclusion, we demonstrated the role of various vitamins, micronutrients, and antioxidants in modifying the risk of developing autoimmune diseases. We highlight the importance of dosage variability when considering prophylactic usage of such supplements, especially for vitamin D. Our study contributes to the evolving landscape of nutritional immunology, providing a foundation for future research to unravel more definite relationships between micronutrients and autoimmune diseases.

## Data Availability

The raw data supporting the conclusions of this article will be made available by the authors, without undue reservation.
