# Supplementary material for: Vitamin, antioxidant and micronutrient supplementation and the risk of developing incident autoimmune diseases: a systematic review and meta-analysis
Source: Front Immunol. 2024 Dec 9;15:1453703. doi: 10.3389/fimmu.2024.1453703 (PMC11663920; doi:10.3389/fimmu.2024.1453703)

**Vitamins, antioxidants or micronutrients supplementation and the risk of developing incident autoimmune diseases: A systematic review, and meta-analysis**

Supplementary Table 1: Search strategy

Supplementary Table 2: Mixed effects meta-regression of vitamin D supplementation against potential effect moderators (continuous and categorical study-level characteristics) for autoimmune disorders
Supplementary Table 3: Sensitivity meta-analyses of vitamin D supplementation and relative risk ratio of autoimmune disorders, based on study-reported dosage using the random effect model

Supplementary Table 4: Meta-analyses of other vitamins, antioxidants or micronutrients and relative risk ratio of autoimmune disorders using the random effect model

Supplementary Table 5: Evaluation of the mediating or confounding effect of Vitamins on autoimmune diseases

Supplementary Table 6: Evaluation of the mediating or confounding effect of Micronutrients on autoimmune diseases

Supplementary Table 7: Evaluation of the mediating or confounding effect of diet on vitamins, antioxidants or micronutrients and autoimmune diseases

Supplementary Table 8: Evaluation of the mediating or confounding effect of personal characteristics variables on vitamins, antioxidants or micronutrients and autoimmune diseases

Supplementary Table 9: Evaluation of the mediating or confounding effect of smoking on vitamins, antioxidants or micronutrients and autoimmune diseases

Supplementary Table 10: Evaluation of the mediating or confounding effect of physical or outdoor activity on vitamins, antioxidants or micronutrients and autoimmune diseases

Supplementary Table 11: Quality assessment of included cohort studies using the Joanna Brigg’s Institute Critical Appraisal tool

Supplementary Figure 1: Funnel plot for visual inspection of publication bias in studies assessing vitamin D supplementation and autoimmune diseases

Supplementary Figure 2: Trim-and-fill analysis for publication bias in studies assessing vitamin D supplementation and autoimmune diseases

Supplementary Figure 3: Quantitative assessment publication bias in studies assessing vitamin D supplementation and autoimmune diseases

Supplementary Figure 4: Leave-one-out analysis of studies assessing vitamin D supplementation and autoimmune diseases

Supplementary Figure 5: Outlier assessment of studies assessing vitamin D supplementation and autoimmune diseases

Supplementary Table 1: Search strategy

| **Database** | **Search terms** | **Results** |
| --- | --- | --- |
| PubMed | ("Micronutrients"[Mesh] OR "Vitamins"[Mesh] OR "Antioxidants"[Mesh] OR "Micronutrient*"[Title/Abstract] OR “Vitamin*”[Title/Abstract] OR "Antioxida*"[Title/Abstract])  AND ("Autoimmune Diseases"[Mesh] OR “Disease, Autoimmune”[Title/Abstract] OR “Disease*, Autoimmune”[Title/Abstract] OR “Autoimmune Disease*”[Title/Abstract] OR “Rheumatoid Arthritis”[Title/Abstract] OR “Psorias*”[Title/Abstract] OR "Thyroid Disease*"[Title/Abstract] OR “Multiple Sclerosis”[Title/Abstract])  AND ('incidence*'[Title/Abstract] OR 'risk*[Title/Abstract] OR 'prevalence*'[Title/Abstract]) | 3,600 |
| EMBASE | ('vitamin supplementation'/exp OR 'vitamin intake'/exp OR 'micronutrient intake'/exp OR 'multivitamin'/exp OR 'vitamin*':ti,ab OR 'antioxidant*':ti,ab)   AND ('autoimmune disease*':ti,ab OR 'auto immune disease*':ti,ab OR 'auto-immune disease*':ti,ab OR 'autoimmune disorder*':ti,ab OR 'auto immune disorder*':ti,ab OR 'auto-immune disorder*':ti,ab OR 'auto immunologic disease*':ti,ab OR 'autoimmunologic disease*':ti,ab OR 'autoagressive disease*':ti,ab OR 'autoagression disease*':ti,ab OR 'autoantibody disease*':ti,ab OR 'rheumatoid arthritis':ti,ab OR 'psoriasis':ti,ab OR 'thyroid disease':ti,ab OR 'multiple sclerosis':ti,ab)   AND ('incidence*':ti,ab OR 'risk*':ti,ab OR 'prevalence*':ti,ab)  AND [Embase]/lim | 4,610 |

Results were limited to Year 2000.

Supplementary Table 2: Mixed effects meta-regression of vitamin D supplementation against potential effect moderators (continuous and categorical study-level characteristics) for autoimmune disorders

|  | **Ratio** | **P** | **95% CI Lower** | **95% CI Upper** | **I^2^ (% residual heterogeneity)** |
| --- | --- | --- | --- | --- | --- |
| Gender>25% | -0.2173 | 0.3211 | -0.6465 | 0.2119 | 74.66% |
| Age>50 | -0.0888 | 0.6823 | -0.5140 | 0.3364 | 76.69% |
| Smoking>30% | -0.1047 | 0.6386 | -0.5413 | 0.3320 | 76.21% |

Abbreviations: CI, confidence interval

Supplementary Table 3: Subgroup meta-analyses of vitamin D supplementation and relative risk ratio of autoimmune disorders, based on study-reported dosage using the random effect model

| **Variable** | **Cohorts** | **Number at risk (Exposed)** | **Number at risk (Controls)** | **Risk Ratio** | **95% CI** | **I2** | **Test of interaction (p-value)** |
| --- | --- | --- | --- | --- | --- | --- | --- |
| Overall | 11 | 65602 | 68553 | 0.88 | 0.77; 1.00 | 29.9% | NA |
| Vit D <200IU | 2 | 17191 | 17914 | 0.89 | 0.54; 1.44 | 0.00% | 0.07 |
| Vit D 200-400IU | 2 | 17191 | 17914 | 0.80 | 0.60; 1.06 | 0.00% |  |
| Vit D 400-600IU | 2 | 17191 | 17914 | 0.81 | 0.51; 1.29 | 0.00% |  |
| Vit D 600-800IU | 1 | 953 | 1717 | 0.55 | **0.38; 0.82** | NA |  |
| Vit D 1000IU | 1 | 49 | 50 | 0.94 | 0.65; 1.37 | NA |  |
| Vit D 2000IU | 1 | 12927 | 12944 | 0.79 | 0.63; 1.01 | NA |  |
| Vit D 5000IU | 1 | 51 | 50 | 1.20 | 0.86; 1.66 | NA |  |
| Vit D 10000IU | 1 | 49 | 50 | 1.17 | 0.84; 1.63 | NA |  |

Abbreviations: NA, Not Available; CI, confidence interval

Supplementary Table 4: Meta-analyses of other vitamins, antioxidants or micronutrients and relative risk ratio of autoimmune disorders using the random effect model

| **Variable** | **Cohorts** | **Number at risk (Exposed)** | **Number at risk (Controls)** | **Risk Ratio** | **95% CI** | **I2** | **Test of heterogeneity (p-value)** |
| --- | --- | --- | --- | --- | --- | --- | --- |
| Vit C | 2 | 355 | 1036 | 0.48 | 0.07; 3.26 | 94.0% | <0.01 |
| Vit E | 2 | 19788 | 20204 | 1.17 | 0.65; 2.10 | 70.0% | 0.07 |
| Iron | 2 | 355 | 1036 | 0.52 | 0.07; 3.75 | 95.0% | <0.01 |
| Vit B | 2 | 355 | 1036 | 0.40 | 0.04; 4.10 | 90.0% | <0.01 |
| Multivit | 2 | 355 | 1036 | 0.89 | 0.69; 1.15 | 35.0% | 0.21 |
| Omega 3 | 3 | 13288 | 13974 | 0.98 | 0.84; 1.14 | 0% | 0.41 |

Abbreviations: CI, confidence interval

Supplementary Table 5: Evaluation of the mediating or confounding effect of vitamins on autoimmune diseases

| **Author** | **Year** | **Country** | **Study population** | **Dosage** | **Key findings†** |
| --- | --- | --- | --- | --- | --- |
| **Vitamin A** | | | | | |
| Costenbader | 2010 | USA | 184,643 females followed up over a total of 23 years as part of the Nurses’ Health Study I and II. | Median of  670 IU/day or  1020 IU/day or  1420 IU/day or  2019 IU/day or  3236 IU/day | No significantly modified risk of developing rheumatoid arthritis across all subgroups of dosage. |
| Abdollahpour | 2022 | Iran | 547 cases 1057 controls, mean age 30.8 (SD 8.2) years, recruited by the Iranian Multiple Sclerosis Society. | NR | Significant reduction in risk of developing multiple sclerosis (OR=0.49, 59%CI: 0.41-0.60). |
| Kronzer | 2022 | USA | 212 cases and 636 controls, combined mean age 64 (SD 14) years, recruited from the Mayo Clinic Biobank database from 2009 to 2015. | NR | No significantly modified risk of developing rheumatoid arthritis (OR=2.75, 95%CI: 0.76-9.95). |
| **Vitamin B** | | | | | |
| Pedersen | 2005 | Denmark | 56,691 individuals between 50 and 64 years of age, followed up over a mean of 5.3 years as part of the Danish Diet, Cancer and Health cohort. | NR | No significantly modified risk of developing multiple sclerosis (RR=1.01, 95%CI: 0.91-1.11). |
| Kronzer | 2022 | USA | 212 cases and 636 controls, combined mean age 64 (SD 14) years, recruited from the Mayo Clinic Biobank database from 2009 to 2015. | NR | No significantly modified risk of developing rheumatoid arthritis (RR=1.71, 95%CI: 0.74-3.93) |
| Rezaeimanesh | 2021 | Iran | 143 cases and 400 controls, mean age 47 (SD 9.57) years and 33.68 (SD 8.38) years respectively, followed up between 2018 and 2019. | NR | Significant reduction in risk of developing multiple sclerosis (RR=0.12, 95%CI: 0.02-0.61). |
| Abdollahpour | 2022 | Iran | 547 cases 1057 controls, mean age 30.8 (SD 8.2) years, recruited by the Iranian Multiple Sclerosis Society. | NR | Significant reduction in risk of developing multiple sclerosis (RR=0.51, 95%CI: 0.43-0.61). |
| **Vitamin C** | | | | | |
| Costenbader | 2010 | USA | 184,643 females followed up over a total of 23 years as part of the Nurses’ Health Study I and II. | Median of  90 mg/day or  141 mg/day or  195 mg/day or  308 mg/day or  720 mg/day | No significantly modified risk of developing rheumatoid arthritis or systemic lupus erythematosus across all subgroups of dosage. |
| Cerhan | 2003 | USA | 35,635 females, mean age 64.1 years, recruited as part of the Iowa Women’s Health Study between 1987 and 1997. | Median of  <145 mg/day or  145-251 mg/day or  >251 mg/day | Significant reduction in risk of developing rheumatoid arthritis for subgroup consuming 145-251 mg/day (RR=0.60, 95%CI: 0.40-0.91). No significant reduction in risk of developing rheumatoid arthritis for the other subgroups of dosage. |
| Kronzer | 2022 | USA | 212 cases and 636 controls, combined mean age 64 (SD 14) years, recruited from the Mayo Clinic Biobank database from 2009 to 2015. | NR | No significantly modified risk of developing rheumatoid arthritis (RR=1.71, 95%CI: 0.74-3.93) |
| Rezaeimanesh | 2021 | Iran | 143 cases and 400 controls, mean age 47 (SD 9.57) years and 33.68 (SD 8.38) years respectively, followed up between 2018 and 2019. | NR | Significant reduction in risk of developing multiple sclerosis (RR=0.08, 95%CI: 0.02-0.27). |
| Abdollahpour | 2022 | Iran | 547 cases 1057 controls, mean age 30.8 (SD 8.2) years, recruited by the Iranian Multiple Sclerosis Society. | NR | Significant reduction in risk of developing multiple sclerosis (RR=0.73, 95%CI: 0.66-0.81). |
| **Vitamin E** | | | | | |
| Pedersen | 2005 | Denmark | 56,691 individuals between 50 and 64 years of age, followed up over a mean of 5.3 years as part of the Danish Diet, Cancer and Health cohort. | NR | No significantly modified risk of developing rheumatoid arthritis (RR=1.51, 95%CI: 0.72-3.91). |
| Costenbader | 2010 | USA | 184,643 females followed up over a total of 23 years as part of the Nurses’ Health Study I and II. | Median of  6 mg/day or  8 mg/day or  11 mg/day or  19 mg/day or  128 mg/day | No significantly modified risk of developing rheumatoid arthritis or systemic lupus erythematosus across all subgroups with varying median amounts of intake between 6 mg/day to 128 mg/day. |
| Kronzer | 2022 | USA | 212 cases and 636 controls, combined mean age 64 (SD 14) years, recruited from the Mayo Clinic Biobank database from 2009 to 2015. | NR | No significantly modified risk of developing rheumatoid arthritis (RR=2.71, 95%CI: 0.84-5.60) |
| Karlson | 2008 | USA | 39,876 females, mean age 54.6 (SD 7) years, recruited as part of the Women’s Health Study, followed up between 1992 and 1995. | NR | No significantly modified risk of developing rheumatoid arthritis (RR=0.89, 95%CI: 0.61-1.31). |
| Cerhan | 2003 | USA | 35,635 females, mean age 64.1 years, recruited as part of the Iowa Women’s Health Study between 1987 and 1997. | 8.6-20.1 IU/day or  more than 20.1 IU/day | No significantly modified risk of developing rheumatoid arthritis across both sub groups with varying median amounts of intake. |
| **Multivitamin** | | | | | |
| Kronzer | 2022 | USA | 212 cases and 636 controls, combined mean age 64 (SD 14) years, recruited from the Mayo Clinic Biobank database from 2009 to 2015. | NR | No significantly modified risk of developing rheumatoid arthritis (OR=1.84, 95%CI: 0.784-4.33). |
| Rezaeimanesh | 2021 | Iran | 143 cases and 400 controls, mean age 47 (SD 9.57) years and 33.68 (SD 8.38) years respectively, followed up between 2018 and 2019. | NR | No significantly modified risk of developing multiple sclerosis (OR=0.67, 95%CI: 0.31-1.42). |

Abbreviations: CI, confidence interval; OR, odds ratio; RR, risk ratio; SD, standard deviation; NR, not reported

†Outcomes of interest include logistic or linear regression analysis for any association between different types of vitamins and risk of autoimmune diseases

Supplementary Table 6: Evaluation of the mediating or confounding effect of micronutrients on autoimmune diseases

| **Author** | **Year** | **Country** | **Study population** | **Dosage** | **Key findings†** |
| --- | --- | --- | --- | --- | --- |
| **Iron** | | | | | |
| Pedersen | 2005 | Denmark | 56,691 individuals between 50 and 64 years of age, followed up over a mean of 5.3 years as part of the Danish Diet, Cancer and Health cohort. | NR | No significantly modified risk of developing rheumatoid arthritis (RR=1.01, 95%CI: 0.91-1.11). |
| Cortese | 2019 | USA | 175,431 women followed up over a total of 23 years as part of the Nurses’ Health Study I and II. | Median of less than 20 mg/day, or  more than 20 mg/day | No significantly modified risk of developing multiple sclerosis across a median intake of less than 20mg/day (RR=1.09, 95%CI: 0.81-1.47) or more than 20mg/day (RR=1.01, 9%CI: 0.73-1.40). |
| Kronzer | 2022 | USA | 212 cases and 636 controls, combined mean age 64 (SD 14) years, recruited from the Mayo Clinic Biobank database from 2009 to 2015. | NR | No significantly modified risk of developing rheumatoid arthritis (RR=1.69, 95%CI: 0.65-4.41). |
| Rezaeimanesh | 2021 | Iran | 143 cases and 400 controls, mean age 47 (SD 9.57) years and 33.68 (SD 8.38) years respectively, followed up between 2018 and 2019. | NR | No significantly modified risk of developing rheumatoid arthritis (RR=0.83, 95%CI: 0.33-2.07). |
| Abdollahpour | 2022 | Iran | 547 cases 1057 controls, mean age 30.8 (SD 8.2) years, recruited by the Iranian Multiple Sclerosis Society. | NR | No significant reduction in risk of developing multiple sclerosis (RR=0.91, 95%CI: 0.83-1.00). |
| **Omega-3** | | | | | |
| Kronzer | 2022 | USA | 212 cases and 636 controls, combined mean age 64 (SD 14) years, recruited from the Mayo Clinic Biobank database from 2009 to 2015. | NR | No significantly modified risk of developing rheumatoid arthritis (OR=1.08, 95%CI: 0.76-1.56) |
| Cortese | 2015 | Norway | 953 cases and 1717 controls, combined mean age 45.5 (SD 10.7) years, recruited as part of the Environmental Factors in Multiple Sclerosis Study. | NR | Significant reduction in risk of developing multiple sclerosis with intake of cod liver oil during adolescence (OR=0.72, 95%CI: 0.55-0.96). |
| Cavalla | 2022 | Italy | 83 cases and 83 controls, mean age 35.38 and 34.31 years respectively, recruited from a single centre in Italy. | NR | Significant reduction in risk of developing a first demyelinating event (OR=0.28, 95%CI: 0.10-0.77) |
| **Calcium** | | | | | |
| Cortese | 2019 | USA | 175,431 women followed up over a total of 23 years as part of the Nurses’ Health Study I and II. | Median of,  1058 mg/day or  1154 mg/day or  1629 mg/day or  1741 mg/day | No significantly modified risk of developing multiple sclerosis across all subgroups with varying median amounts of intake. |
| Racovan | 2011 | USA | 32435 postmenopausal female participants of mean age 62.3 years, SD 6.92, were enrolled in the Women’s Health Initiative Calcium plus Vitamin D trial. | NR | No significantly modified risk of developing rheumatoid arthritis (HR =1.04, 95%CI: 0.76-1.41) |
| Kronzer | 2022 | USA | 212 cases and 636 controls, combined mean age 64 (SD 14) years, recruited from the Mayo Clinic Biobank database from 2009 to 2015. | NR | No significantly modified risk of developing rheumatoid arthritis (OR=1.09, 95%CI: 0.74-1.61) |
| Abdollahpour | 2022 | Iran | 547 cases 1057 controls, mean age 30.8 (SD 8.2) years, recruited by the Iranian Multiple Sclerosis Society. | NR | Significant reduction in risk of developing multiple sclerosis (RR=0.49, 95%CI: 0.39-0.63). |
| **Zinc** | | | | | |
| Cortese | 2019 | USA | 175,431 women followed up over a total of 23 years as part of the Nurses’ Health Study I and II. | Median of,  14 mg/day or  15.6 mg/day or  26.3 mg/day or  1741 mg/day | No significantly modified risk of developing multiple sclerosis across all subgroups with varying median amounts of intake. |
| Pedersen | 2005 | Denmark | 56,691 individuals between 50 and 64 years of age, followed up over a mean of 5.3 years as part of the Danish Diet, Cancer and Health cohort. | NR | No significantly modified risk of developing rheumatoid arthritis (RR=0.99, 95%CI: 0.93-1.05) |
| Kronzer | 2022 | USA | 212 cases and 636 controls, combined mean age 64 (SD 14) years, recruited from the Mayo Clinic Biobank database from 2009 to 2015. | NR | No significantly modified risk of developing rheumatoid arthritis (OR=1.59, 95%CI: 0.75-3.34) |
| Cerhan | 2003 | USA | 35,635 females, mean age 64.1 years, recruited as part of the Iowa Women’s Health Study between 1987 and 1997. | Less than 15 mg/day or  more than 15.5 mg/day | Significant reduction in risk of developing rheumatoid arthritis in the subgroup taking more than 15.5mg/day (RR=0.39, 95%CI: 0.17-0.88). |
| Abdollahpour | 2022 | Iran | 547 cases 1057 controls, mean age 30.8 (SD 8.2) years, recruited by the Iranian Multiple Sclerosis Society. | NR | Significant reduction in risk of developing multiple sclerosis (RR=0.34, 95%CI: 0.28-0.42). |
| **Dietary total antioxidant index** | | | | | |
| Moradi | 2022 | Iran | 100 cases and 197 controls, mean age 43.7 (SD 11.4), recruited across a single centre in Iran. | NR | Significant reduction in risk of developing rheumatoid arthritis with second tertile (OR=0.14, 95%CI: 0.04-0.51) and third tertile (OR=0.28, 95%CI: 0.10-0.76) of dietary total antioxidant capacity. |
| Abdollahpour | 2022 | Iran | 547 cases 1057 controls, mean age 30.8 (SD 8.2) years, recruited by the Iranian Multiple Sclerosis Society. | NR | Significant reduction in risk of developing multiple sclerosis with higher dietary antioxidant index (OR=0.84, 95%CI: 0.80-0.89) |

Abbreviations: CI, confidence interval; OR, odds ratio; RR, risk ratio; SD, standard deviation; NR, not reported

†Outcomes of interest include logistic or linear regression analysis for any association between different types of vitamins and risk of autoimmune diseases

Supplementary Table 7: Evaluation of the mediating or confounding effect of diet on vitamins, antioxidants or micronutrients and autoimmune diseases

| **Author** | **Year** | **Country** | **Study population** | **Key findings†** |
| --- | --- | --- | --- | --- |
| Pederson | 2005 | Denmark | 57053 participants of mean age 57 years, SD 7, residing in Copenhagen or Aarhus were recruited by the Civil Registration System in Denmark. | Medium fat fish (3-7 g fat/100 g fish) was associated with significantly increased risk of RA (p<0.05). Intake of fruit and coffee was not associated with risk of RA. |
| Kronzer | 2022 | USA | 100 participants with rheumatoid arthritis and 300 age matched controls of mean age 64 year, SD NR, were recruited within Mayo Clinic Biobank, mainly from Mayo Clinic primary care locations in Minnesota. | Three or more servings high-fat food and 5+ servings  fruits/vegetables daily showed non-significant associations with RA (aOR 1.22, 95% CI 0.74–2.00 vs. 0–1 time;  aOR 0.75, 95% CI 0.51–1.11 vs. 0–3 times). |
| Cavalla | 2022 | Italy | 83 cases and 83 controls, mean age 35.38 and 34.31 years respectively, recruited from a single centre in Italy. | Higher intake of rapid absorption carbohydrates, lower intake of vegetal proteins, and higher intake of animal proteins were observed in patients with a first demyelinating event (P<0.05). |
| Rezaeimanesh | 2021 | Iran | 143 cases and 400 controls, mean age 47 (SD 9.57) years and 33.68 (SD 8.38) years respectively, followed up between 2018 and 2019. | More intake of dairy (OR: 0.27; 95%CI: 0.14-0.53), seafood (OR: 0.21; 95%CI: 0.10-0.44), red meat (OR:0.44; 95%CI: 0.22-0.90), vegetable (OR: 0.19; 95%CI: 0.09-0.39), fruit (OR: 0.47; 95%CI: 0.22-0.99) and nut (OR: 0.29; 95%CI: 0.15-0.56) in the third tertiles resulted in significant reduction in MS risk. |
| Abdollahpour | 2022 | Iran | 547 cases of incident multiple sclerosis and 1057 population controls of mean age 30.8 years, SD 8.2 were recruited by the Iranian Multiple Sclerosis Society. | Significantly decreased risk of developing multiple sclerosis with egg and red meat intake in grams/week (OR: 0.95, 95%CI: 0.92-0.97), poultry intake in serves/week (OR: 0.96, 95%CI: 0.52-0.80) and dietary supplement intake (OR: 0.90, 95%CI: 0.84-0.96). |
| Dehghan | 2018 | Iran | 120 patients with multiple sclerosis and 360 healthy controls, mean age 30.9 years, SD 3.51, recruited from three educational hospitals and one multiple sclerosis charity association in Kerman, southeastern Iran. | Significantly increased risk of developing multiple sclerosis for carnivorous diets (OR:11.78, 95%CI: 5.10-27.19) as compared to vegetarian diets (OR:10.05, 95%CI: 2.60-38.84). |
| Merlino | 2004 | USA | 29368 women of mean age 61.5, SD 4.2 were recruited from the Iowa Women’s Health Study and followed up with self-administered questionnaires. | Significantly decreased risk of developing rheumatoid arthritis for intake of more than 68 servings of milk products in a month (RR: 0.63, 95%CI: 0.41-0.98). Intake of butter, margarine, skim milk and whole milk alone was not associated with risk of rheumatoid arthritis. |

Abbreviations: SD, standard deviation; OR, odds ratio; CI, confidence interval; RA, rheumatoid arthritis; MS, multiple sclerosis

†Outcomes of interest include logistic or linear regression analysis for any association between diet and risk of autoimmune diseases

Supplementary Table 8: Evaluation of the mediating or confounding effect of personal characteristics variables on vitamins, antioxidants or micronutrients and autoimmune diseases

| **Author** | **Year** | **Country** | **Study population** | **Key findings†** |
| --- | --- | --- | --- | --- |
| Butzkeuven | 2023 | Australia | 204 participants with a new onset of first ever clinical episode of CNS demyelination of mean age 37 years, SD 10.3 were recruited from 23 academic multiple sclerosis centres across Australia and New Zealand | Significantly increased risk of developing multiple sclerosis per one year increase in age (HR: 0.97, 95%CI: 0.95-0.99).  No significantly modified risk of developing multiple sclerosis based on sex, (p=0.42), skin color (p>0.10), BMI (p>0.10) and disability level (EDSS score) (p>0.10). |
| Racovan | 2011 | USA | 32435 postmenopausal female participants of mean age 62.3 years, SD 6.92, were enrolled in the Women’s Health Initiative Calcium plus Vitamin D trial. | Significantly increased risk of developing rheumatoid arthritis per one year increase in age (p=0.03). |
| Dehghan | 2018 | Iran | 120 patients with multiple sclerosis and 360 healthy controls, mean age 30.9 years, SD 3.51, recruited from three educational hospitals and one multiple sclerosis charity association in Kerman, southeastern Iran. | Significantly increased risk of developing multiple sclerosis in the population with family income of <357 dollars (OR:1.48, 95%CI: 2.41-8.00) as compared to population with family income of >357 dollars. |
| Hahn | 2021 | USA | 25871 participants of mean age 67.1 years, SD 7.1 were recruited throughout the USA. | Significantly increased efficacy of omega-3 fatty acid in preventing autoimmune diseases in participants with a family history of autoimmune disease (HR: 0.66, 95%CI:0.43-0.99) as compared to participants with no family history of autoimmune disease. |

Abbreviations: SD, standard deviation; OR, odds ratio; RR, risk ratio; CI, confidence interval; HR, hazard ratio; EDSS, expanded disability status scale

†Outcomes of interest include logistic or linear regression analysis for any association between personal characteristics variables and risk of autoimmune diseases

Supplementary Table 9: Evaluation of the mediating or confounding effect of smoking on vitamins, antioxidants or micronutrients and autoimmune diseases

| **Author** | **Year** | **Country** | **Study population** | **Key findings†** |
| --- | --- | --- | --- | --- |
| Abdollahpour | 2022 | Iran | 547 cases of incident multiple sclerosis and 1057 population controls of mean age 30.8 years, SD 8.2 were recruited by the Iranian Multiple Sclerosis Society. | No significantly modified risk of developing multiple sclerosis based on tobacco (OR: 1.20, 95%CI: 0.75-1.96) or waterpipe smoking (OR: 1.35, 95%CI: 0.99-1.83).  Significantly increased risk of developing multiple sclerosis with lifetime second hand smoking (OR: 1.86, 95%CI: 1.51-2.29). |
| Pedersen | 2005 | Denmark | 57053 participants of mean age 57 years, SD 7, residing in Copenhagen or Aarhus were recruited by the Civil Registration System in Denmark. | Significantly increased risk of developing rheumatoid arthritis for current smokers (RR: 2.7, 95%CI: 1.48-4.96) as compared to former smokers (RR: 1.63, 95%CI: 0.81-3.29). |
| Cavalla | 2022 | Italy | 83 cases and 83 controls, mean age 35.38 and 34.31 years respectively, recruited from a single centre in Italy. | Ever smoker status (OR 4.472, p = 0.003) were independently associated with risk of  a first demyelinating event. |

Abbreviations: SD, standard deviation; OR, odds ratio; RR, risk ratio; CI, confidence interval

†Outcomes of interest include logistic or linear regression analysis for any association between smoking and risk of autoimmune diseases

Supplementary Table 10: Evaluation of the mediating or confounding effect of physical or outdoor activity on vitamins, antioxidants or micronutrients and autoimmune diseases

| **Author** | **Year** | **Country** | **Study population** | **Key findings†** |
| --- | --- | --- | --- | --- |
| Abdollahpour | 2022 | Iran | 547 cases of incident multiple sclerosis and 1057 population controls of mean age 30.8 years, SD 8.2 were recruited by the Iranian Multiple Sclerosis Society. | Significantly decreased risk of developing multiple sclerosis with physical activity during adolescence amounting to >4000 MET/week (OR: 0.59, 95%CI: 0.45-0.78) as compared to physical activity during adolescence amounting to 2000-4000 MET/week (OR: 0.80, 95%CI: 0.62-1.05).  Significantly decreased risk of developing multiple sclerosis with higher cumulative sunlight exposure during adolescence.  For ½-2h a day: (OR: 0.51, 95%CI: 0.36-0.72),  For 2-3h a day: (OR: 0.32, 95%CI: 0.22-0.47),  For 3-5h a day: (OR: 0.26, 95%CI: 0.18-0.37),  For >5h a day: (OR: 0.19, 95%CI: 0.12-0.30). |
| Kronzer | 2022 | USA | 100 participants with rheumatoid arthritis and 300 age matched controls of mean age 64 years, SD NR, were recruited within Mayo Clinic Biobank, mainly from Mayo Clinic primary care locations in Minnesota. | Significantly increased risk of developing rheumatoid arthritis with increasing active work activity. Active work activity (OR: 3.00, 95%CI: 1.58-5.69), moderate work activity (OR: 2.83, 95%CI: 1.39-5.77.  No significantly modified risk of developing rheumatoid arthritis with leisure physical activity (OR: 0.96, 95%CI: 0.64-1.42). |
| Dehghan | 2018 | Iran | 120 patients with multiple sclerosis and 360 healthy controls, mean age 30.9 years, SD 3.51, recruited from three educational hospitals and one multiple sclerosis charity association in Kerman, southeastern Iran. | Significantly increased risk of developing multiple sclerosis with sun exposure surface of only face and two hands (OR: 2.08, 95%CI:1.24-3.15). |

Abbreviations: SD, standard deviation; OR, odds ratio; CI, confidence interval; NR, not reported; MET, metabolic equivalent of task

†Outcomes of interest include logistic or linear regression analysis for any association between physical or outdoor activity and risk of autoimmune diseases

Supplementary Table 11: Quality assessment of included cohort studies using the Joanna Brigg’s Institute Critical Appraisal tool

| **Study** | **1** | **2** | **3** | **4** | **5** | **6** | **7** | **8** | **9** | **10** | **11** |
| --- | --- | --- | --- | --- | --- | --- | --- | --- | --- | --- | --- |
| Dehghan | Y | Y | Y | Y | Y | Y | Y | Y | Y | Y | Y |
| Butzkueven | Y | Y | Y | Y | Y | Y | Y | Y | Y | Y | Y |
| Cavalla | Y | Y | Y | Y | Y | Y | Y | Y | Y | Y | Y |
| Hahn | Y | Y | Y | Y | Y | Y | Y | Y | Y | Y | Y |
| Karlson | NA | NA | Y | Y | Y | Y | Y | Y | Y | Y | Y |
| Abdollahpour | Y | Y | Y | Y | Y | Y | Y | Y | Y | Y | Y |
| Cerhan | NA | NA | Y | Y | Y | Y | Y | Y | Y | Y | Y |
| Cortese 2015 | Y | Y | Y | Y | Y | Y | Y | N | N | Y | Y |
| Kronzer | Y | Y | Y | Y | Y | Y | Y | Y | Y | Y | Y |
| Merlino | NA | NA | Y | Y | Y | Y | Y | Y | Y | Y | Y |
| Munger | NA | NA | Y | Y | Y | Y | Y | Y | Y | Y | Y |
| Pedersen | NA | NA | Y | Y | Y | Y | Y | Y | Y | Y | Y |
| Racovan | Y | Y | Y | N | N | Y | Y | Y | Y | Y | Y |
| Rezaeimanesh | Y | Y | Y | Y | Y | Y | Y | NA | NA | NA | Y |
| Cortese 2019 | NA | NA | Y | Y | Y | Y | Y | Y | Y | Y | Y |
| Costenbader | NA | NA | Y | Y | Y | Y | Y | Y | Y | Y | Y |
| Hiraki | NA | NA | Y | Y | Y | Y | Y | Y | Y | Y | Y |
| Moradi | Y | Y | Y | Y | Y | Y | Y | Y | Y | Y | Y |

| Checklist |
| --- |
| 1. Were the two groups similar and recruited from the same population? |
| 2. Were the exposures measured similarly to assign people to both exposed and unexposed groups? |
| 3. Was the exposure measured in a valid and reliable way? |
| 4. Were confounding factors identified? |
| 5. Were strategies to deal with confounding factors stated? |
| 6. Were the groups/participants free of the outcome at the start of the study (or at the moment of exposure)? |
| 7. Were the outcomes measured in a valid and reliable way? |
| 8. Was the follow up time reported and sufficient to be long enough for outcomes to occur? |
| 9. Was follow up complete, and if not, were the reasons to loss to follow up described and explored? |
| 10. Were strategies to address incomplete follow up utilized? |
| 11. Was appropriate statistical analysis used? |

Legend:

Y – Yes

N – No

U – Unclear

NA – Not applicable

Supplementary Figure 1: Funnel plot for visual inspection of publication bias in studies assessing vitamin D supplementation and autoimmune diseases


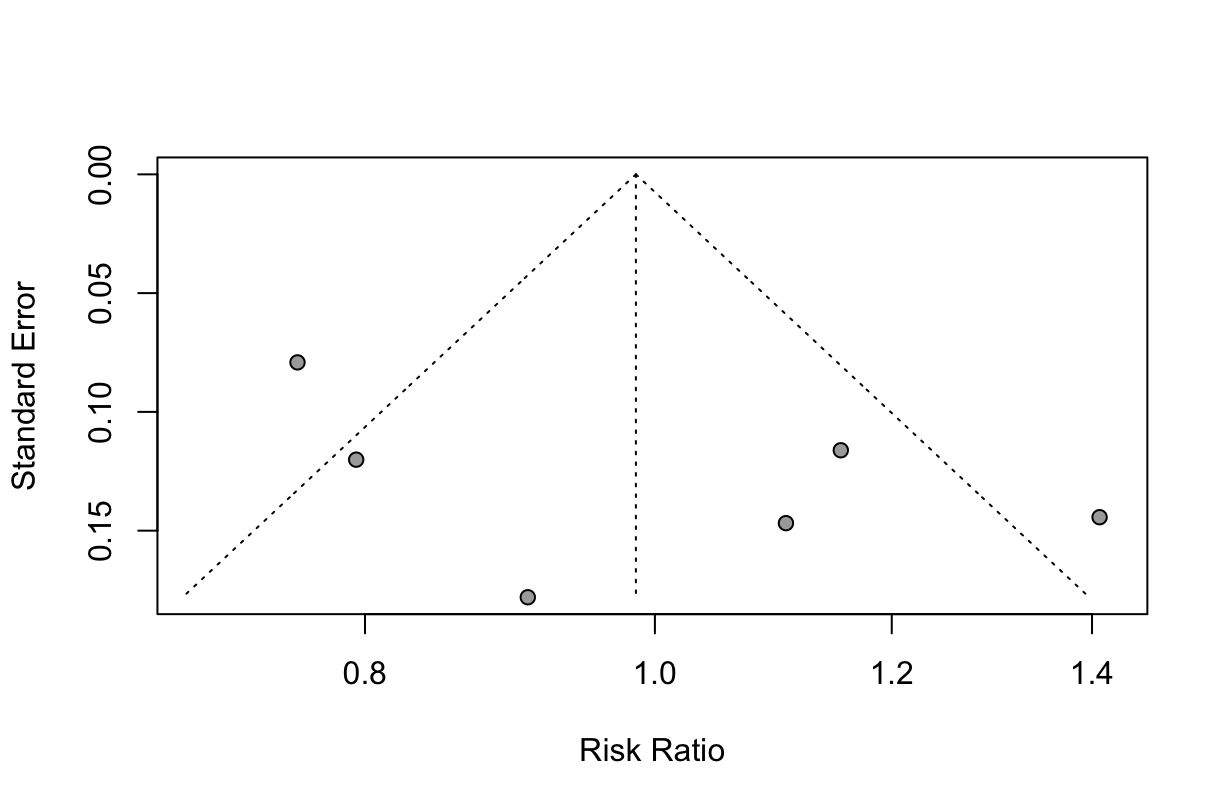


Supplementary Figure 2: Trim-and-fill analysis for publication bias in studies assessing vitamin D supplementation and autoimmune diseases


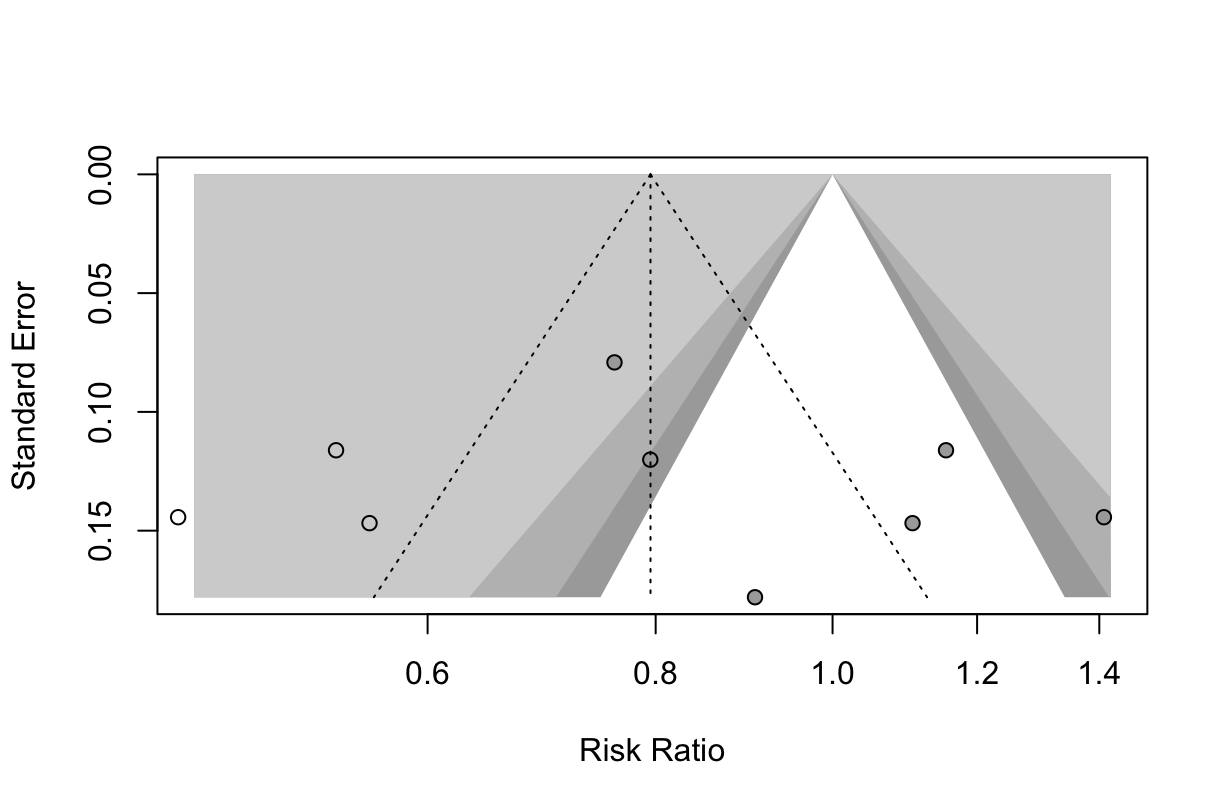


Supplementary Figure 3: Quantitative assessment publication bias in studies assessing vitamin D supplementation and autoimmune diseases


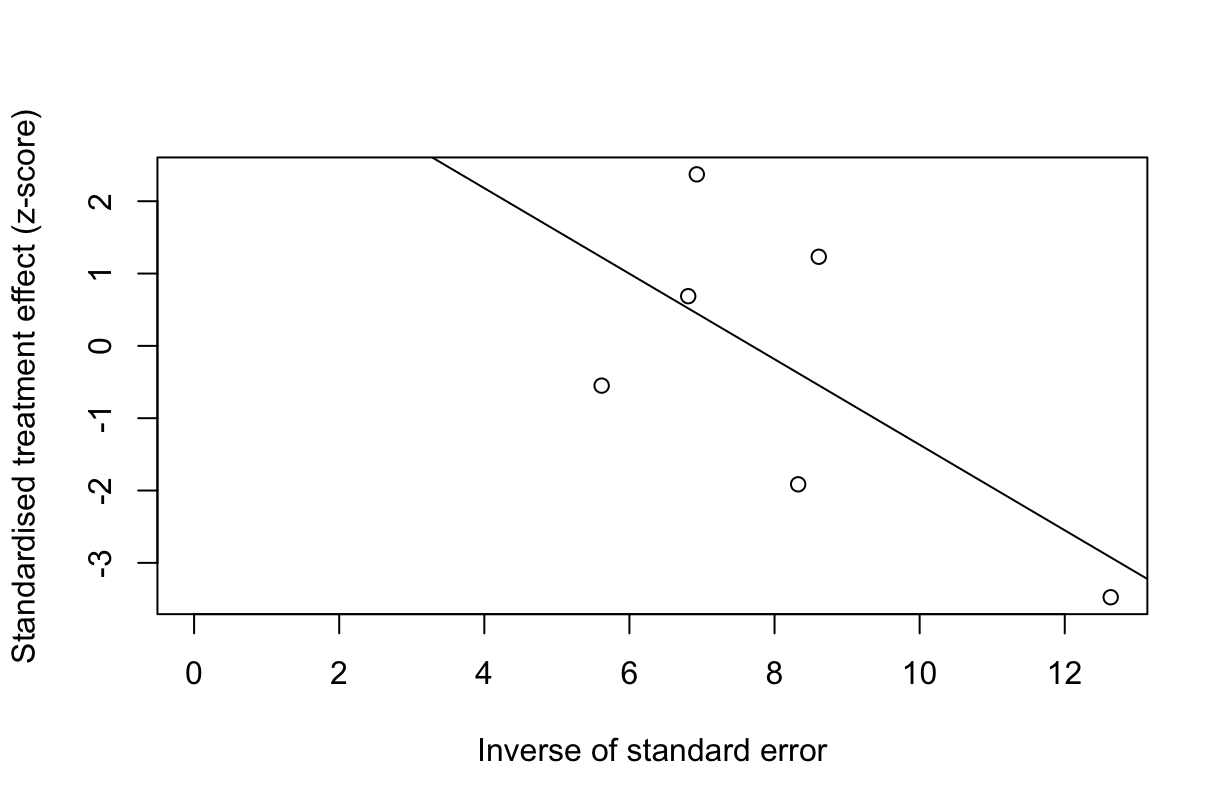


Supplementary Figure 4: Leave-one-out analysis of studies assessing vitamin D supplementation and autoimmune diseases


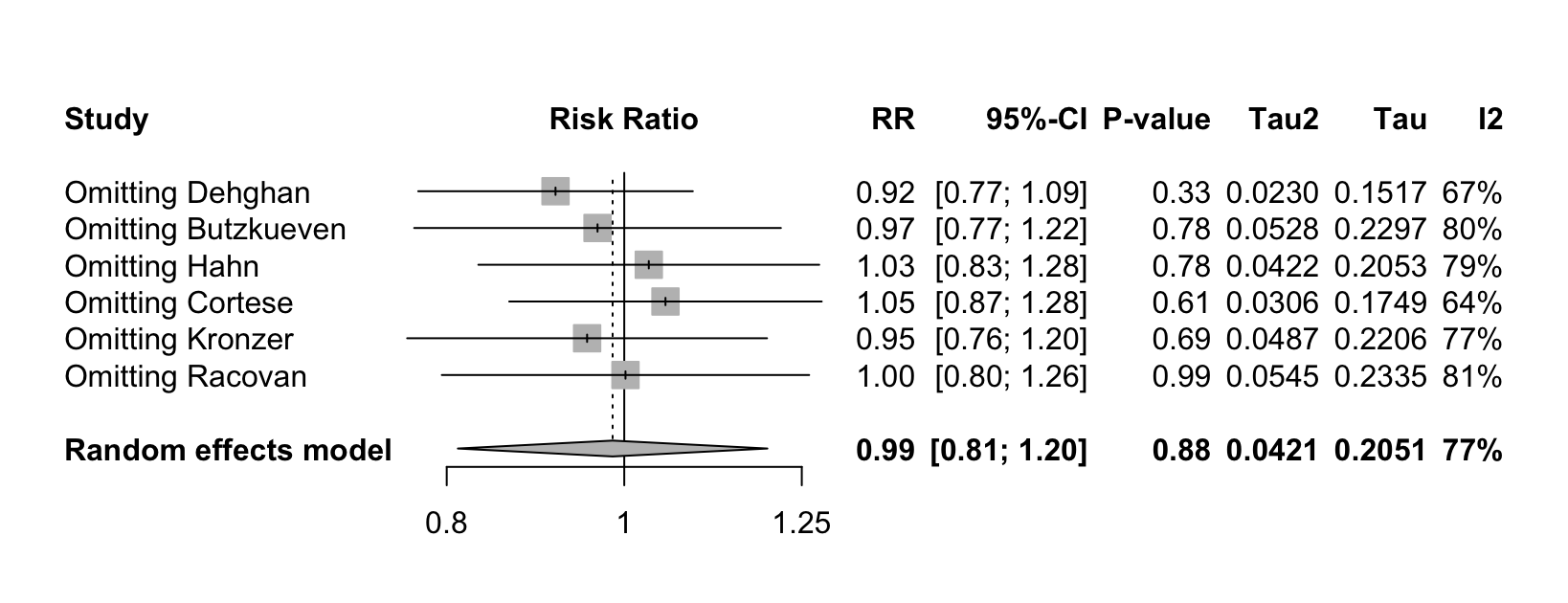


Supplementary Figure 5: Outlier assessment of studies assessing vitamin D supplementation and autoimmune diseases


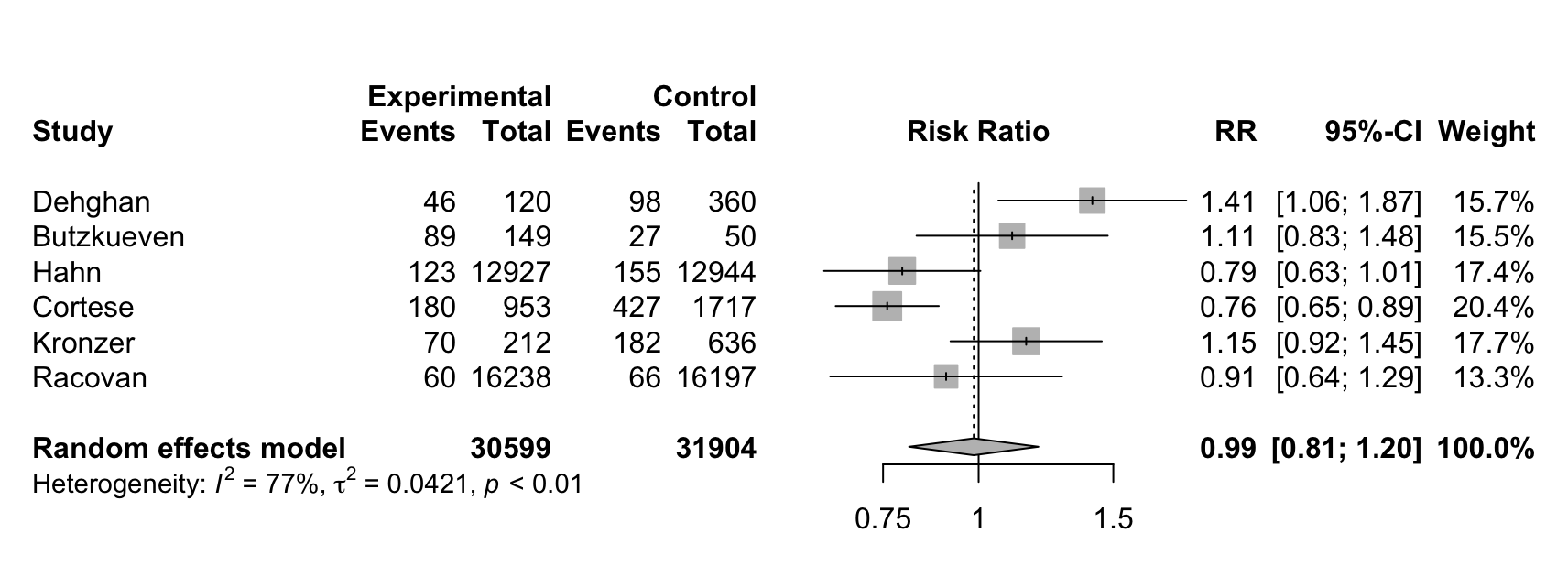

Supplement: Supplementary file 1 [file DataSheet1.docx]
